# A brief history of the Feulgen reaction

**DOI:** 10.1007/s00418-024-02279-9

**Published:** 2024-04-12

**Authors:** Marco Biggiogera, Margherita Cavallo, Claudio Casali

**Affiliations:** Cell Biology and Neurobiology Laboratory, Department of Biology and Biotechnology “Lazzaro Spallanzani”, University of Pavia, Via A.Ferrata 9, 27100 Pavia, Italy

**Keywords:** Feulgen reaction, Schiff-type reagent, Osmium ammine, Light microscopy, Electron microscopy

## Abstract

One hundred years ago, Robert Feulgen published a landmark paper in which he described the first method to stain DNA in cells and tissues. Although a century has passed since the discovery by Feulgen and Rossenbeck, the chemical reaction still exerts an important influence in current histochemical studies. Its contribution in diverse fields, spanning from biomedicine to plant biology, has paved the way for the most significant studies that constitute our current knowledge. The possibility to specifically explore the DNA in cell nuclei while quantifying its content makes it a contemporary and timeless method. Indeed, many histocytochemical studies following the 1924 paper have led to a deep understanding of genome organization in general as well as several specific mechanisms (e.g. DNA duplication or tumour pathology) that, nowadays, constitute some of the most fundamental pillars in biological investigations. In this review, we discuss the chemistry and application of the Feulgen reaction to both light and electron microscopy.

## Introduction

The year 1924 marked a significant milestone in the advancement of histocytochemical studies. That was the year when Feulgen and Rossenbeck published a chemical reaction which allowed the specific staining of deoxyribonucleic acid (DNA) in histological specimens (Feulgen and Rossenbeck 1924). At that time, Watson and Crick’s studies on the structure of the DNA double helix were still far in the future and the scientific community had divergent opinions regarding the presence of DNA within cell nuclei. Although acknowledging the studies of Miescher who had demonstrated the presence of a phosphorous-rich acid presumably present in the nuclei of purulent cells (Miescher 1871), Feulgen and Rossenbeck themselves raised questions about the actual nuclear presence of nucleic acids, recognizing the difficulties arising when isolating them from complex tissues since there was no guarantee that the extracted material actually came from nuclei. Central to their inquiry was the concept behind the meaning of chromatin, which was a term originally employed by histologists to refer to anything within the nucleus that could be stained or coloured (*chromos* in Greek, meaning colour), yet it lacked precise delineation of the underlying chemical properties. The necessity to provide clear answers to these questions led them to the development of a chemical reaction which allowed nucleic acids to be specifically stained within the nucleus. A pivotal role was played by Kossel and Steudel who initially characterized the nitrogenous composition of what was then referred to as thymonucleic acid owing to its thymine content. It was observed that after its cleavage, for instance with nitric acid, it was capable of reducing Fehling’s solution thus resulting in the splitting of purine bases (Jones and Austrian 1907; Leven 1922; Jones 1953). Taking this information into consideration, Feulgen and Rossenbeck devised a method involving a first acid hydrolysis step that allowed the separation of purine bases thereby exposing free aldehyde groups in the DNA structure. These aldehydes could then selectively react with the Schiff reagent, also known as basic fuchsin, which confers a purple-like coloration to the sample. The peculiarity of this reaction is the fact that it specifically stained the DNA as they observed that only thymonucleic acid-containing samples gave rise to a coloured product (Feulgen and Rossenbeck 1924).

It is also important to mention the involvement of Frieda Feulgen, who gave an impactful contribution to the understanding of this chemical reaction; indeed she discussed her doctoral dissertation, entitled “Untersuchungen über die Nuklealfärbung” (Investigations of Nuclear Staining), in 1924, the same year in which Robert Feulgen published his work (Benedeum and Mesch 1999).

Since then, the Feulgen reaction has significantly contributed to various fields spanning from plant biology to human biomedicine elucidating numerous unanswered questions. Considering the influential scientific legacy that the Feulgen reaction brought with it, here we discuss the impact of this cytohistochemical method, highlighting the ongoing significance and utility of this technique which remains one of the most widely used in biology.

## Chemical insights of the Feulgen reaction

**Fig. 1:**
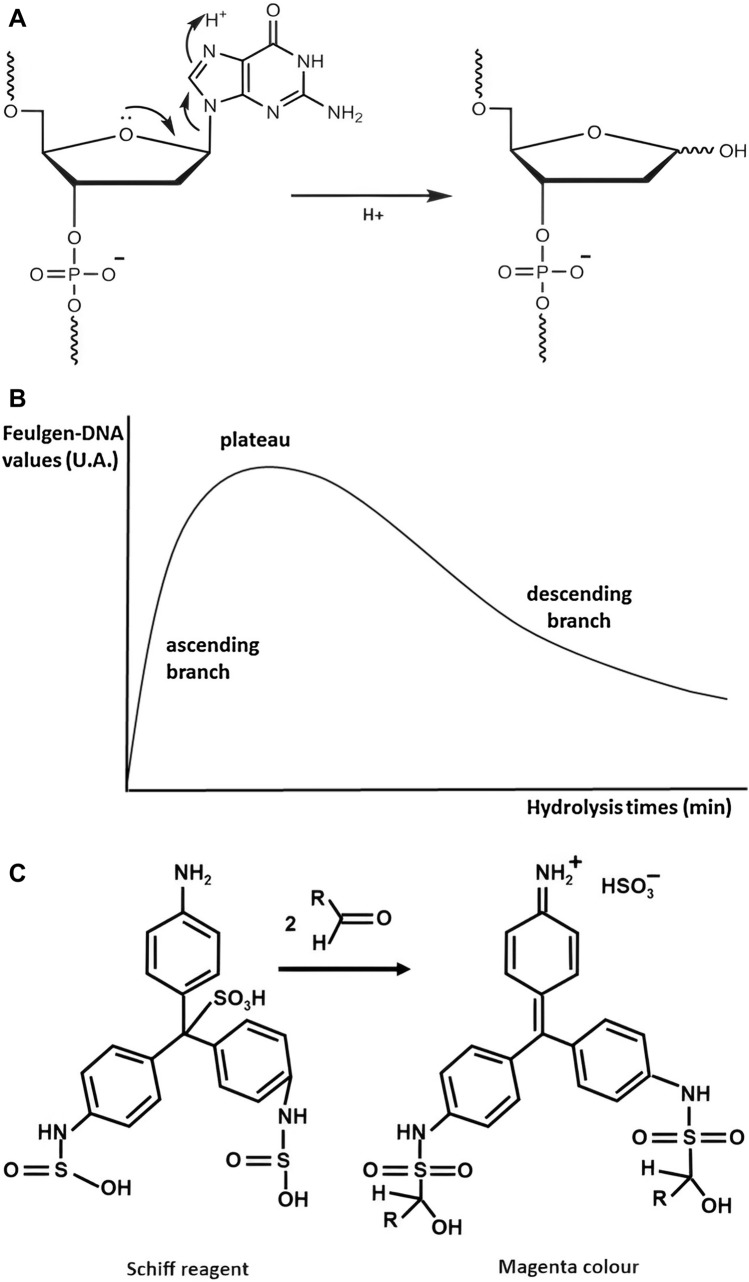
**a** Schematic representation of a DNA nucleotide in an acidic environment. Hydrogen ions mediate the detachment of guanine, therefore leaving the DNA nucleotide apurinated (readapted from Pourshahian 2021). **b** Typical Feulgen hydrolysis curve. The x-axis represents the time required for hydrolysis and the y-axis indicates the Feulgen–DNA values. The plateau corresponds to the maximum level of DNA depurination while the ascending and descending branches represent DNA depurination and depolymerisation, respectively (modified from Mello and Vidal 2017). **c** One of the possible interpretations of the chemical reaction occurring when the Schiff reagent interacts with aldehyde groups. According to this hypothesis, the aldehydic group forms an alkylsulfonic acid when interacting with SO_2_ groups; the binding of the C atom from the alkyl group with the primary aromatic amine re-establishes the chromophoric function of the dye, consequently producing a magenta staining (modified from Hubbe et al. 2019)

A key step in the Feulgen reaction is the detachment of purines from the deoxyribose sugar during the initial acid hydrolysis step (Fig. 1a), thus unmasking free aldehydic groups on the DNA backbone which becomes apurinic. Exposed aldehydes specifically react with the Schiff reagent whose main component consists of bleached pararosaniline that acquires a magenta colour upon DNA binding. Importantly, the Feulgen reaction is specific for DNA staining since RNA is characterized by a ribose sugar, which possesses an –OH group at the 2′ position, preventing acid hydrolysis and thus impeding purine detachment (Chieco and Derenzini 1999).

### Acid hydrolysis

The original method proposed by Feulgen and Rossenbeck (1924) consisted of a treatment with 1 N HCl at 60 °C for 4 min. However, the kinetics of acid hydrolysis depends on multiple factors such as acid concentration, time and temperature, making the initial step of this method crucial for achieving optimal yields. Indeed, as shown in Fig. 1b, as hydrolysis progresses the value along the ascending branch of the curve increases, suggesting a detachment of purine bases from the DNA. Following a plateau stage, which indicates the maximum level of DNA depurination, the curve displays a descending trend indicating DNA breakdown and solubilization as reviewed by Mello and Vidal (2017). Consequently, several histocytochemical studies have focused on exploring different variables that could potentially influence and optimize this initial phase. For instance, in 1972, Andersson and Kjellstrand demonstrated how, although both acid concentration and temperature influenced the reaction speed, changes in HCl concentration corresponded to distinct purine extraction rates (Andersson and Kjellstrand 1972). Five years later, Kjellstrand evaluated the exposure and removal of aldehydes during Feulgen acid hydrolysis by comparing a wide range of temperatures (between 9 and 75 °C) and acid concentrations (from 6 to 0.05 M). From these findings, Kjellstrand concluded that the traditional procedure should be replaced with higher HCl concentrations and lower temperatures (e.g. 5 N HCl at room temperature) (Kjellstrand 1977).

An additional factor that might impact and interfere with acid hydrolysis is the type of fixative employed. Feulgen initially recommended the use of a sublimate-acetic fixative since he considered other fixatives containing oxidizing compounds potentially capable of damaging the nuclear material. In 1939 Hillary noted that the duration of hydrolysis depended on the type of fixative. He observed that chromic acid did not interfere with the staining process; however, a considerably longer period for acid hydrolysis was required whereas fixation with acetic-ethanol required less (Hillary 1939). A decade later, Swift suggested fixation with formaldehyde since he observed a higher yield compared to acetic-ethanol fixed specimens (Swift 1950). However, subsequent groups found that acetic-ethanol fixatives yield the maximum intensity in plant materials (Sharma and Sharma 1980).

### Schiff stain

The second phase of the Feulgen reaction consists of exposing the sample, whether fresh or fixed, to the Schiff reagent (Fig. 1c). The latter, derived from basic fuchsin, is composed of a pararosaniline dye, a member of the triaminotriphenylmethane dye family characterized by three phenyl groups attached to a central carbon atom (Delamater et al., 1950). The Schiff reagent is prepared by bubbling SO_2_ through a 0.5% solution of pararosaniline chloride until saturation, causing sulfur dioxide to bind to the central carbon atom or chromophoric structure, altering its structure and forming a sulfonic acid compound, thereby bleaching the dye. Over time other compounds have been explored as a source of SO_2_, for instance potassium metabisulfite (Na_2_S_2_O_5_) (Kasten 1960; Chieco and Derenzini 1999).

**Fig. 2:**
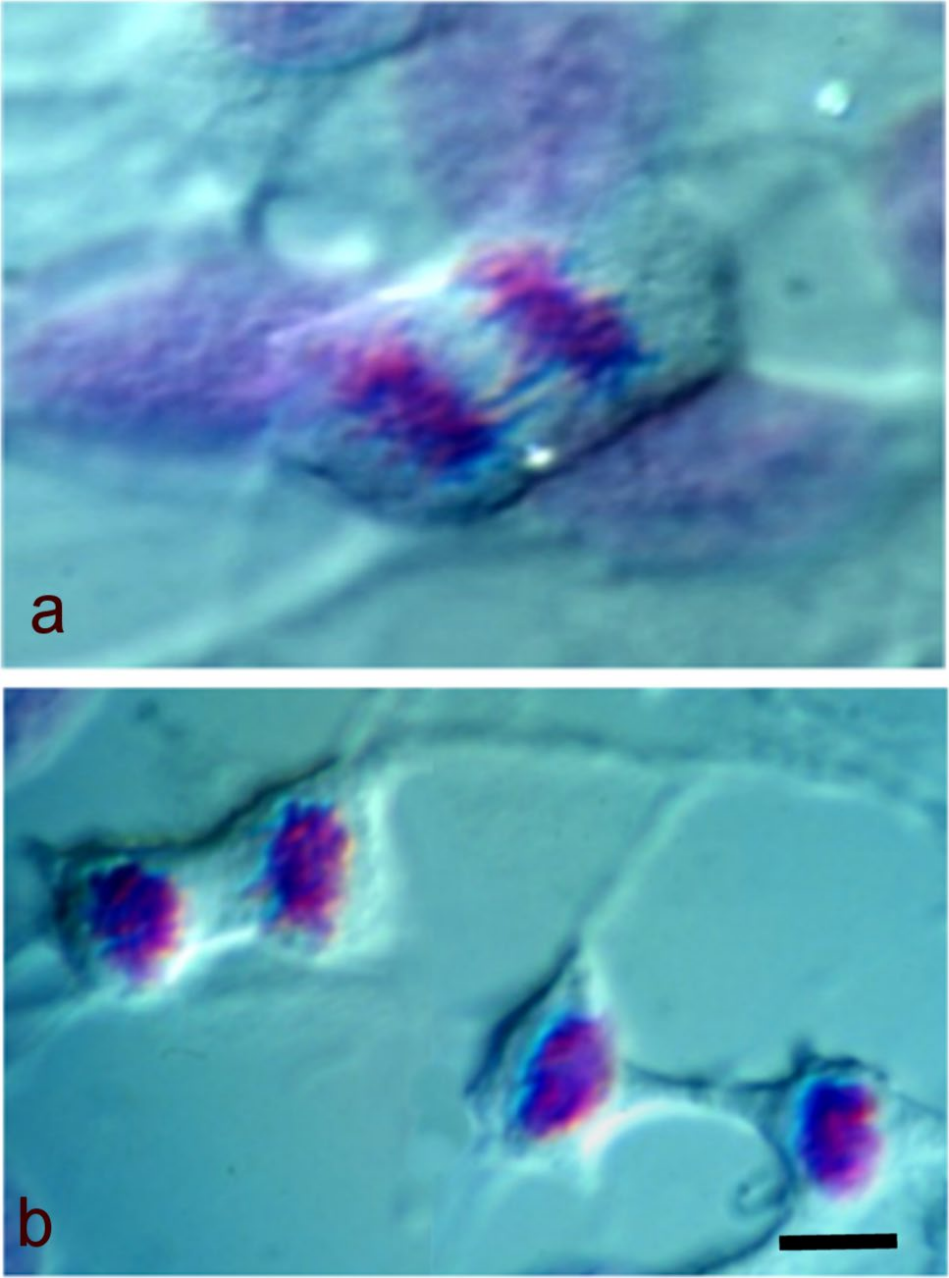
HeLa cells in mitosis after Feulgen reaction and observed under phase contrast. **a** Anaphase; **b** Telophase and late telophase. Courtesy of Carlo Pellicciari. Bar = 10 µm

Upon binding with apurinic acid aldehydes, the reagent assumes its characteristic colour, staining the DNA magenta (Fig. 2). However, numerous theories have emerged regarding the mechanism of this reaction, and its chemical behaviour remains a subject of ongoing debate. The most widely accepted hypothesis, as discussed by Puchtler et al. in 1975, suggests that SO_2_ groups, upon reacting with aldehydes, form alkylsulfonic acid, wherein the carbon atom of the alkyl group binds to the nitrogen atom of the pararosaniline primary aromatic amine, thereby restoring the chromophoric function (Puchtler et al. 1975). These findings were consistent with those reported at the turn of the twentieth century by Prud’homme, who demonstrated that the reaction products of basic fuchsin, sodium bisulfite and formaldehyde are alkylated and sulfonated derivatives of the parent compound (Prud’homme, 1900).

## DNA quantitative analysis

Despite the debate over the dynamics of the chemical reaction, Feulgen’s method remained one of the principal means used to specifically detect and quantify DNA. Nowadays, it is well established that the final colour density is proportional to the number of aldehyde groups on DNA (Kasten 1959; Dujindam and Van Duijn 1975). Initially, however, there was scepticism in this regard, e.g. Lessler questioned the reliability of a DNA cytochemical quantification as hypothesized by Widström and Caspersson years before (Widström, 1928; Caspersson 1936). However, the criticism did not stop the observations of Ris and Mirsky, who, in 1949, demonstrated that the DNA content is approximately constant within nuclei of one species (Mirsky and Ris 1949). They evaluated the intensity of the reaction by absorption measurements with the optical microscope and compared the data obtained with those acquired with a spectrophotometer confirming their results (Lessler 1948; Mirsky and Ris 1949; Swift 1950). Therefore, following Mirsky and Ris’s work, DNA content investigation became a widely used tool to explore cell and nuclear mechanisms. For instance, Patau and Swift demonstrated that cells double their DNA content prior to mitosis and provided insightful observations concerning the changes occurring in chromosome structures throughout mitosis, thus paving the way for all the studies that have led to our current understanding of the cell cycle (Patau and Swift 1953). DNA quantitative analyses through colour measurements became widely employed in both light microscopy and spectrophotometric analyses leading to interesting investigations. For instance, Salisbury et al. in 1961 noted a decrease in the DNA colour density of bovine spermatozoa upon aging; Cunningham and colleagues investigated the DNA content in nuclei of normal and neoplastic rat tissues finding a significant increase in the second condition (Salisbury et al. 1961; Cunningham et al. 1950). Notably, these analyses had an impact on epigenetic studies and significantly contributed to provide new insights into heterochromatin and euchromatin features. In this regard, Mello explored DNA organization in Malpighian tubes of *Triatoma infestans* by observing spectral profiles of the specimens which exhibited a shoulder at *λ* = 530 nm. The prominent values were associated with heterochromatin areas due to a higher amount of repetitive DNA. Higher signal intensity in heterochromatin areas was due to the proximity of Schiff base molecules which disubstitute free aldehyde groups that are close to each other. This approach paved the way for the investigation of purine base proximity in chromatin regions (Mello 1978).

## More than historical remnants: a brief selection of recent Feulgen reaction applications

Despite being a now-distant milestone in the field of histochemical research and being a foundational method in the field of nuclear cell biology, considering its potential in nucleic acid staining and quantitation, the Feulgen reaction is still being utilized for the modern investigation of multiple facets of DNA and, consequently, cell nuclei. For instance, in a study of hepatocarcinogenesis, Takimoto and colleagues recently demonstrated the formation of Feulgen-positive cytoplasmic inclusions following methylcarbamate exposure, indicative of potential chromosomal instability (Takimoto et al. 2024). In 2023, Alkan and Koroglu-Aydin took advantage of the Feulgen stain of oral epithelium cells to define the histopathological and genotoxic effects of smoking and periodontitis, focusing on DNA damage and micronuclei formation (Alkan and Koroglu-Aydin 2023). Similarly, in 2022, Paiva and colleagues combined the Feulgen reaction with other histochemical stains in the study of oral mucosa exposed to carcinogens (Paiva et al. 2022). In addition, Kobayashi et al. investigated nuclear profiles in lung carcinoma using the Feulgen reaction, with particular attention given to the quantitation of the nuclear DNA content (Kobayashi et al. 2019).

These examples of recent applications clearly show the timelessness and impact of this foundational histochemical technique. However, although the main field reported here is associated with human biology, particularly cancer research, it is important to remark that the Feulgen reaction has crossed barriers between different biological areas. Concerning wider outlooks, Fidler and Gomes combined the qualitative and quantitative results of the Feulgen reaction for precise estimation of chromatin condensation and cellular rearrangements of the cerebellar layers in X-ray-exposed mice (Fidler and Gomes 2023). Furthermore, other recent works not specifically focused on human biology have exploited the DNA specificity of the Feulgen reaction. We could not even speculate whether Robert Feulgen would have imagined that his technique could have been applied to characterize and distinguish between two different drosophilids, *Zaprionus indianus* and *Zaprionus sepsoides*, species which differ in the size of their testes and can be told apart from each other by the spermatogenesis-applied Feulgen reaction (de Almeida Rego et al. 2013). Focusing on other animal cell investigations, Feulgen staining was applied to evaluate several nuclear parameters, such as shape and size, DNA content, and chromatin compaction, in lymphocytes and epithelial cells of canine origin (Dos Santos et al. 2019). Similar morphometric aspects were also investigated by Giuliano and colleagues in llama sperm nuclei, with specific attention dedicated to chromatin distribution and measurement of the haploid DNA content (Giuliano et al. 2018).

Not only animal but also plant cell research has relied on the Feulgen reaction to perform specific DNA analysis. Indeed, in 2020, the application of Feulgen staining was described for imaging of ovaries and developing embryo sacs in maize (Kalinowska et al. 2020). During the same year, Wojtczak applied the Feulgen reaction to study the spermatid differentiation of the alga *Chara vulgaris*, highlighting important similarities with mammal spermiogenesis (Wojtczak 2020).

These examples show that the Feulgen reaction represents a valid tool in various biological applications, ranging from animal to plant cell biology, even in the 2020s—a hundred years after its first description. Remarkably, the Feulgen reaction even found relevance during the COVID-19 pandemic, when it was used to investigate nuclei of mucosa samples collected from patients with COVID-19 (Sadik et al. 2023).

## The quest for a Feulgen-type reagent for electron microscopy

The Italian and, foremostly, the Pavia School of Histochemistry have been fertile ground for the usage and advancement of the Feulgen reaction. A significant improvement was the development of novel, more stable, and easily reproducible Schiff-type reagents, aiming to overcome limitations such as the intrinsic difficulties inherent in completing the laborious synthesis steps. These innovations allowed the Feulgen reaction to efficiently expand its histochemical applications into areas such as flow cytometry, where it served for quantitative DNA determination for both research and clinical purposes, and in particular electron microscopy (EM), granting DNA visualization with ultrastructural resolution (Casali et al. 2022; Mazzini 2024).

Interestingly, although the Feulgen reaction has been known since 1924, it took almost 50 years before finding a reagent capable of satisfying the requirements for its use in EM. This looks like a paradox, but the reasons were substantial. First a Feulgen-type reagent should have at least one ammino group and an electrondense core to be visible by EM. Schiff’s reagent, in fact, is only very weakly electrondense (Gautier 1976). The obvious steps to take were to (a) increase artificially Schiff electrondensity or (b) completely change the reagent type.

As for the first approach, many attempts were made (Gautier 1976), but the most promising was proposed by Moyne (1973) utilizing thallium ethylate. The procedure, however, was quite long, involving en bloc acetylation to block hydroxyl groups, en bloc hydrolysis, and then finally Schiff staining and thallium counterstaining on thin sections. Moreover, thallium is a highly poisonous metal. These factors, together with the fact that the specimens prepared with this procedure were only suitable for DNA staining and nothing else (thus preventing different stainings on adjacent sections), finally doomed the technique.

As for alternative approaches to adapt the Feulgen reaction to EM, a few attempts must be mentioned. The very first involved the reduction of silver atoms where aldehydes were present. Already in 1924 Feulgen and Volt had shown that after a mild hydrolysis, silver ions could precipitate on the newly generated aldehyde groups on DNA (Feulgen and Volt 1924). Breitschneider (1949) first and Peters (1966) later stained whole sperm heads or thin sections for EM analysis. Silver, unfortunately, can also precipitate where SH-rich proteins are, for instance, or where other silver ions are present. The reaction, hence, was definitely interesting but only preferential and not specific (Hayat 1993).

A completely different attempt to solve this important problem was the NAMA-Ur procedure (Testillano et al. 1991). This method involved en bloc staining and a long series of passages and resulted in a highly contrasted end product. Although suffering from the same drawbacks as thallium, it has been rediscovered for serial sectioning and reconstruction of an entire cell nucleus stained for DNA using this approach has been reported (Roquette et al. 2009).

**Fig. 3:**
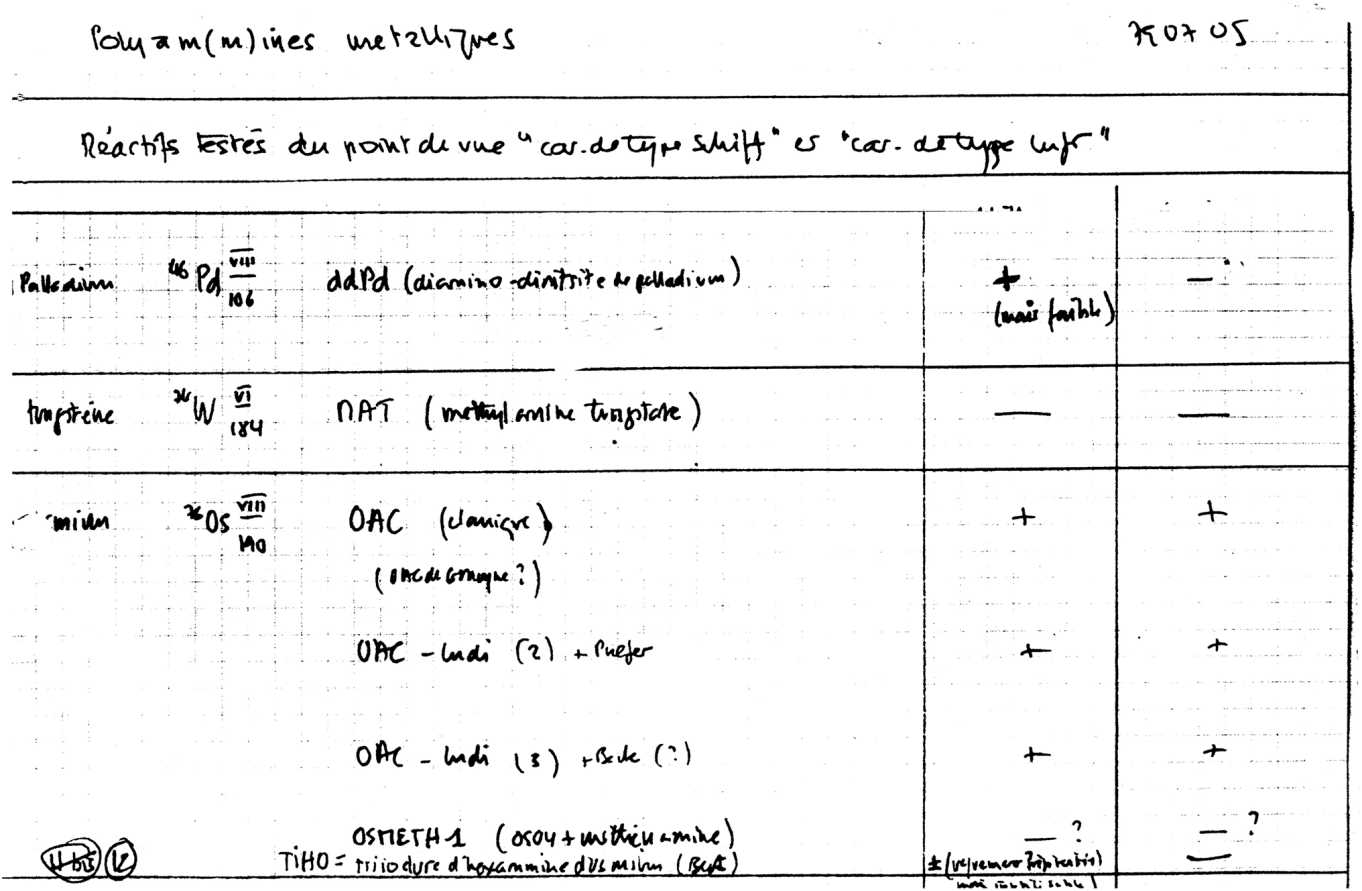
From Alain Gautier’s archives. The text reads: Metallic polyam(m)mines. Reagents tested for a Schiff-type or Luft-type specificity. Diamino dinitrite palladium; methylamine tungstate; osmium ammine complexes. As shown, palladium and tungsten are weakly positive or negative, while osmium ammine results were positive in all the different batches utilized. The exception is represented by Osmeth (osmium methenammine) and hexaammineosmium triiodide

The most difficult way (and, finally, the successful one) was the search for a new electrondense Schiff-type reagent. While working at the Centre de Microscopie Electronique (CME) of the University of Lausanne, I (M.B.) had the opportunity to meet Alain Gautier and look through his archives of notebooks. For several years he carefully recorded the numerous compounds he tested in order to find the right reagent, including Bismarck brown, hexammineruthenium, hexachloroosmate etc. (Fig. 3). The great majority were discarded, but finally the CME group was able to synthesize an osmium-based polyammine with Schiff-type character. The reagent was called osmium ammine complex (OA) (Cogliati and Gautier 1973). This paper was the first to report a specific Feulgen-type reaction for EM. The results were remarkable: specific, extremely fine-grained, high resolution, useful to stain DNA as well as in PAS-type reactions (periodic acid–Schiff, for polysaccharides), perfect for thin sections.

A minor problem was its low contrast; a major problem was its long and costly synthesis with few well-defined steps and many steps linked to the change in colour of the solution. Briefly, the synthesis worked in about 40–50% of the cases, and after a certain period it was impossible to reproduce it in Lausanne, but it worked in the Derenzini lab, in Bologna. Olins and coworkers (1989) eventually described a new type of synthesis of OA which was then called OA-B. The synthesis was reproducible and the reagent became commercially available. So far, however, the chemical formula of neither OA nor OA-B has been clarified or provided.

## Applications of osmium ammine

The first papers that applied the Feulgen reaction to EM (Cogliati and Gautier 1973; Gautier et al. 1973) were mostly dedicated to the demonstration of the Schiff-type qualities of OA. Only later came the biological applications. One of the first to embrace the technique was Moyne (Moyne et al. 1978; Moyne 1980), who analysed the virus-induced nuclear inclusions, revealing viral DNA before the appearance of other nuclear alterations.

**Fig. 4:**
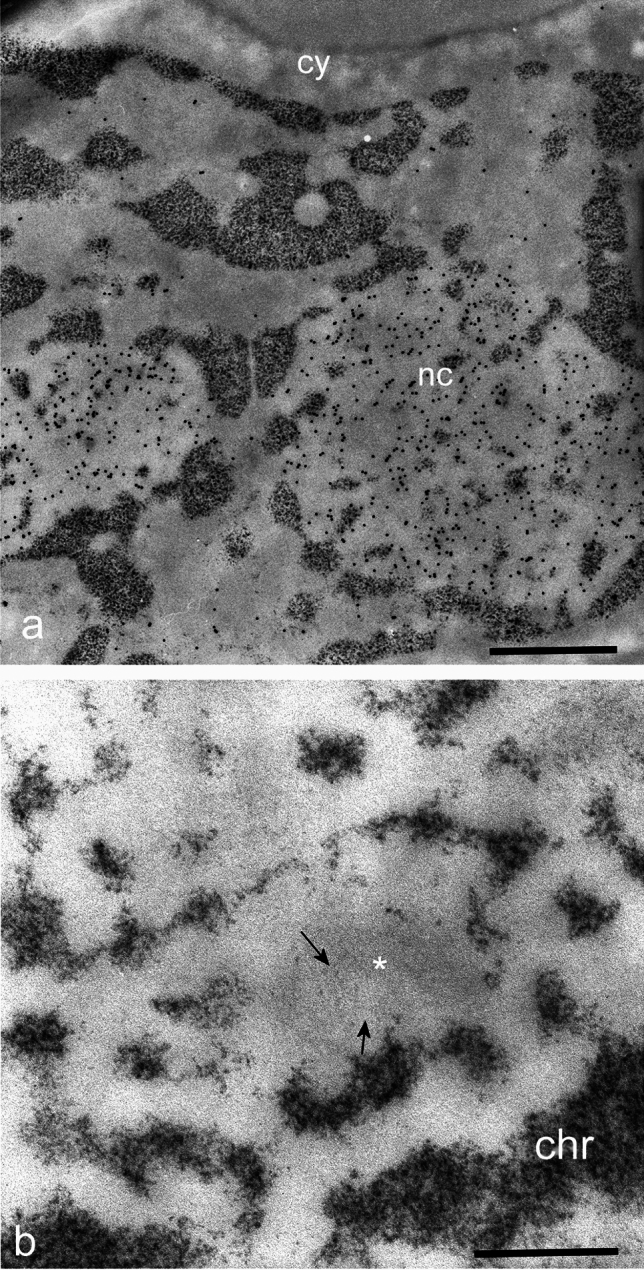
**a** P815 mouse mastocytoma cell, immunolabelled for nucleolin and stained for DNA with OA-B. HCl hydrolysis 45 min, OA-B staining 60 min. Note the intense nucleolar labelling and the highly contrasted DNA. cy: cytoplasm; nc: nucleolus. Bar = 500 nm. **b** P815 cell, OA-B staining. At high magnification note the fibrillar centre (asterisk) and thin filament of DNA emerging from the condensed DNA region the region. chr: condensed chromatin at the periphery of the nucleolus. Bar = 100 nm

Most of the interesting papers came from the group of Derenzini. In several publications they showed the structure of chromatin in situ in the nucleus (Derenzini et al. 1982, 1983) at the level of the thinnest chromatin fibres. In a couple of papers (Hernandez-Verdun et al. 1982; Hernandez-Verdun and Derenzini 1983) they studied the structure of chromatin inside the nucleolus, describing the presence of areas of DNA without histones, in a non-nucleosomal conformation. The high resolution of the technique allowed them to visualize individual nucleosomes, highlighting nucleosomal DNA surrounding an electron-lucent histone core (Hernandez-Verdun and Derenzini 1983) (Fig. 4).

Detection of DNA within individual mitochondria as well as in viruses was shown to be feasible (Liu et al. 1992; Puvion-Dutilleul et al. 1996).

Another important application was the visualization of the S-phase distribution of DNA in parallel with immunolabelling for BrdU which demonstrated the possibility to utilize OA after other cytochemical procedures (el-Alfy et al. 1995). Indeed, the technique can be carried out after immunolabelling provided that the thin sections are left to dry for some time. Acid hydrolysis does not displace or remove colloidal gold particles, thus allowing precise localization of both DNA and the target proteins (Biggiogera et al. 2001). OA was also proven to work perfectly on cryosections (Puvion and Bernhard 1975).

Interestingly, detection of DNA at the EM level showed some strange patterns in particular types of chromatin. In mature sperm nuclei in mouse (Biggiogera 1986) and other species (Courtens et al. 1991, 1994), the cell nucleus, homogeneously stained after exposure to uranyl and lead, becomes stained in spots, as if the DNA was unevenly distributed. The reason for this behaviour lies in microheterogeneities of the protamine–protamine interaction (Boutinard Rouelle-Rossier and Biggiogera 1992) rather than reflecting biological features. Furthermore, chromosomes and their characteristics were also studied with interesting findings related to their structure (Liu et al. 1995). For example, developing mouse embryos (Fakan and Odartchenko 1980) were shown to exhibit a thin rim of DNA surrounding the prenucleolar bodies. The stained DNA then “colonized” the entire nucleolus. Several reviews summarize in depth the wide spectrum of applications of OA in revealing chromatin architecture (Biggiogera et al. 1996; Derenzini et al. 2014; Biggiogera 2024).

## Future perspectives

By tracing the historical development and widespread adoption of the Feulgen reaction across various scientific domains, its enduring relevance in many still evolving fields becomes evident. To date, many approaches based on the Feulgen reaction have been developed, most directed towards gaining a comprehensive understanding of chromatin organization through the evaluation of geometric, densitometric and textural features. By integrating diverse tools such as light microscopy and cytometry it has been possible to assess chromatin condensed areas among different organisms or conditions, exploring changes in chromatin density during different developmental stages, aging progression, and tumorigenesis (for a review, see Mello and Vidal 2017). However, the Feulgen method, when supported by microscopy-based approaches, offers the possibility to compare morphological data with absorption analyses, therefore enabling the specific observation of variations in DNA distribution across nuclei and its intensity. Comparison of these parameters between distinct specimens offers the opportunity to investigate between numerous chromatin organization patterns, therefore providing unexplored insights into epigenetics, a still expanding field of research.

## Data Availability

No datasets were generated or analysed during the current study.
